# Aging Immune System and Its Correlation With Liability to Severe Lung Complications

**DOI:** 10.3389/fpubh.2021.735151

**Published:** 2021-11-23

**Authors:** Yongtao Li, Chengfei Wang, Meilian Peng

**Affiliations:** ^1^State Key Laboratory for Diagnosis and Treatment of Infectious Diseases, The First Affiliated Hospital, College of Medicine, Zhejiang University, Hangzhou, China; ^2^Department of Maternity, Zhejiang Provincial People's Hospital, Hangzhou, China

**Keywords:** aging immunity, immunosenescence, mortality, lung complications, lymphocytes

## Abstract

Aging is considered to be a decline in physical and physiological events that extensively affect the body's immunity, and is linked with deterioration in both innate and adaptive immune responses. The immune system exhibits profound age-associated variations, known as immunosenescence, comprising a significantly low production of B and T lymphocytes in bone marrow and thymus, a decreased function of mature lymphocytes in secondary lymphoid tissues, a decrease in the synthesis of fresh naïve T cells, and reduced activation of T cells. Elderly individuals face a greater risk for many diseases particularly respiratory diseases due to their poor response to immune challenges as vigorously as the young. The current review explored the aging immune system, highlight the mortality rates of severe lung complications, such as pneumonia, COVID-19, asthma, COPD, lung cancer, IPF, and acute lung injury, and their correlation with aging immunity. This study can be helpful in better understanding the pathophysiology of aging, immune responses, and developing new approaches to improve the average age of the elderly population.

## Introduction

Health systems around the world are facing major challenges due to global demographic fluctuations. In both the developed and developing worlds, a reduced mortality rate has enhanced life expectancy (1). The resulting shift in age demographics necessitates innovative ways to ensure that longer life spans do not come at the cost of aging populations' life quality. The worsening impact of aging on organ and system activity, as well as the intrinsic and extrinsic elements of the environment that influence the progression of this deterioration, must all be considered in new approaches (2). Aging is marked by a decline in physical as well as physiological events that considerably affect the body's defense system. The immune system undergoes a variety of changes with advancing age, eventually losing the ability to mount an effective cellular defense against infection (3). Immunosenescence is a multifactorial phenomenon that affects both arms of the immune system and can be greatly affected by genetic factors as well as extrinsic factors like food, physical activities, co-morbidities, physical and mental stress, prior exposure to microorganisms, toxins, and prescribed medications (4–7).

Research continues to discover approaches to overcoming age-related declines in immunity. Novel approaches to specifically target the immune system as well as geroscience-directed approaches, which target the underlying causes of aging, should provide us with exciting new therapies to extend the healthspan of older adults. Several approaches are already in use or are undergoing testing. First, vaccines specifically formulated for older adults are now available and have been shown to provide greater protection from viral infections such as influenza (8). These vaccines have higher concentrations of antigen or are formulated with adjuvants to boost aging immune responses (8). Second, approaches that alter metabolic activities such as treatment with metformin (9) or mTOR inhibitors (10) could help improve immunity and resistance to infectious diseases in older adults. Last, senolytics, which are a novel class of drugs that target the destruction of senescent cells, have been shown to alleviate age-related diseases in animal models and could also improve aged immune function (11).

## Aging and Adaptive Immunity

The adaptive immune system deteriorates rapidly with age and is the most common problem among the elderly. Immunity relies on T and B cells producing a vast repertoire of antigen receptors, followed by activation and clonal proliferation (12). The stimulation of adaptive immunity is dependent not only on the identification of a specific antigen receptor but also on key signals provided by the innate immune system. The decrease of *de-novo* production of T and B cells is a well-known age-associated immune system modification (13).

B lymphocytes' principal purpose is to produce specific antibodies against a specific pathogen's infection. The B cell arm of the adaptive immune system undergoes considerable changes with aging (14). Delayed B cell responses and ineffective antibodies generation are common in aging individuals, resulting in a diminished capacity to efficiently respond to viral and bacterial pathogens (15). According to the reported studies, the formation of antibodies in response to vaccination against hepatitis B virus infection was considerably lower in 61-year-old donors compared to 33-year-old donors (16). The population of memory B lymphocytes IgG^+^ IgD^−^ CD27^−^, double negative, DN increases in the aged people with a characteristic inflammatory microenvironment. IgG^+^ IgD^−^ CD27^−^ DN B cells, particularly, have a tissue trafficking phenotype and can be induced to generate granzyme-B. DN cells are believed to be exhausted B cells that produce high levels of inflammatory cytokines, such as TNF-α, IL-1, and IL-6. Increased systemic levels of pro-inflammatory cytokines are associated with aging. Multiple studies have found a chronic mild inflammation in aging, described as “inflame-aging,” which may enhance age-related complications (17).

**Figure 1:**
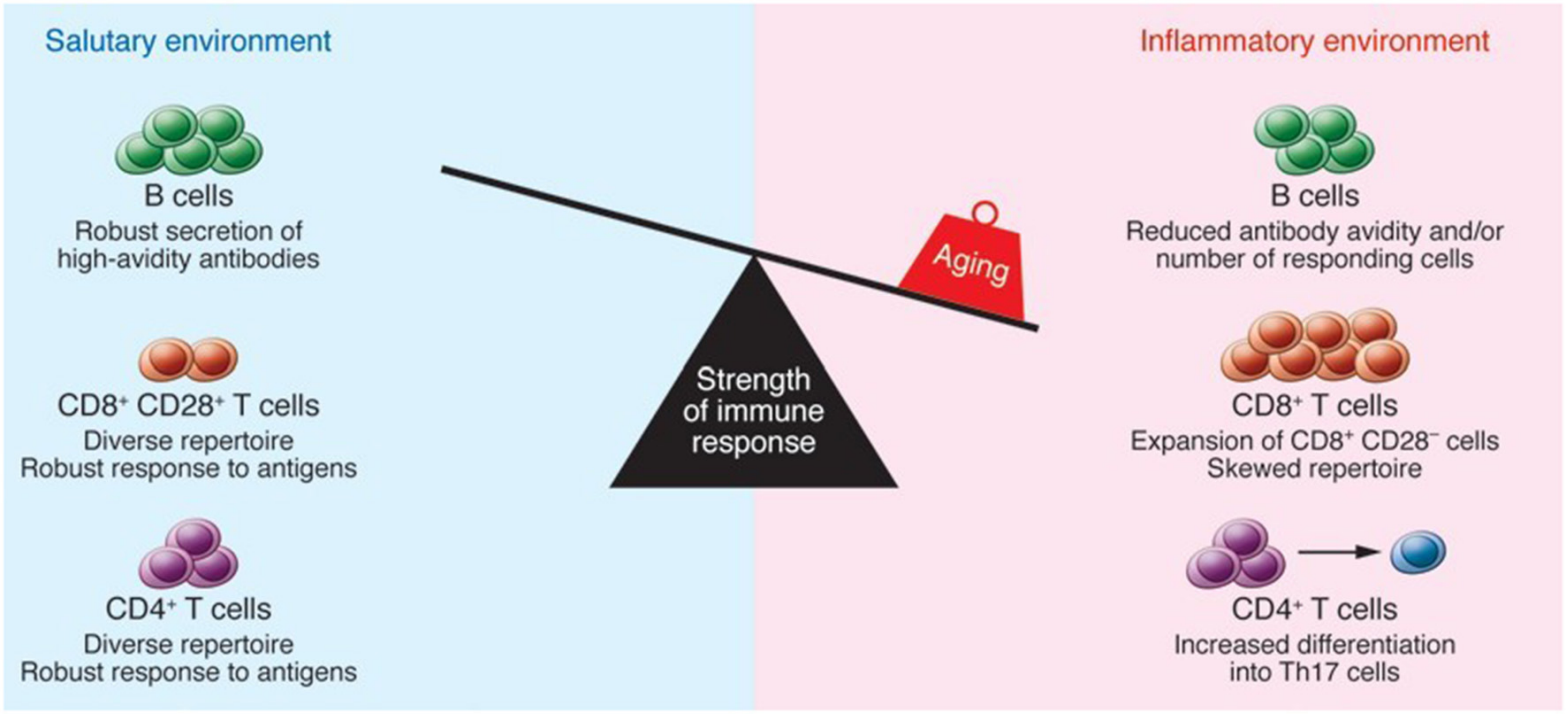
The impact of aging on immune function. Reproduced with permission from Montecino-Rodriguez et al. (22).

T cells are considered to be one of the main constituents of the adaptive immune system. T cells play a crucial role in devastating infected host cells, triggering other immune cells, generating cytokines, and regulating immune reactions. In the context of major histocompatibility complex (MHC), these cells can be separated into CD4 and CD8 detecting antigens (15). Regression of the thymus (age-associated) has been linked with the decreased production of naïve T cells. The underlined phenomenon may results in the decreased T cell diversity in aged people that lead to the higher susceptibility to autoimmune diseases and infectious disorders (18). With increasing age, chronic antigenic load and oxidative stress lower the vulnerability of lymphocytes to damage-induced cell death and raise proinflammatory state, resulting in greater activation of induced-cell death (19). The lymphocyte composition and function in secondary lymphoid tissues can be influenced by a variety of age-associated alterations. CD4^+^ Th cells show greater conversion into Th17 cells. Furthermore, older CD4^+^ Th cells have been observed with several activation abnormalities. In humans, CD8^+^ T cells expand oligoclonally and lose CD28, resulting in poor function. Antibody avidity in response to carbohydrate antigens is lowered, and the number of B cells that act against influenza is reduced. Inflammatory cytokines, which may be produced by stroma, dendritic cells (DCs), or aged B and T lymphocytes are also present in higher concentrations in the tissue microenvironment (20). The elevated number of memory cells occupying tissue niches, as well as the inflammatory site, may make it more difficult to migrate naive B and T cells from the thymus and bone marrow to settle in the tissue. Hence, an increase in memory T cells that have lost CD28 expression is associated with a decrease in immunity which is thought to be a key feature of senescent T cells. *In vitro* experiments have shown that loss of telomerase activity occurs concurrently with loss of CD28 expression in T cells, indicating that CD28 signaling is required for optimal telomerase up-regulation. Senescent cells frequently change their gene expression, making them less prone to apoptosis, and many secrete molecular factors like cytokines, which influence their microenvironment (21). The immune function of aged individuals is weakened as a result of the underlined alterations as represented in Figure 1.

**Figure 2:**
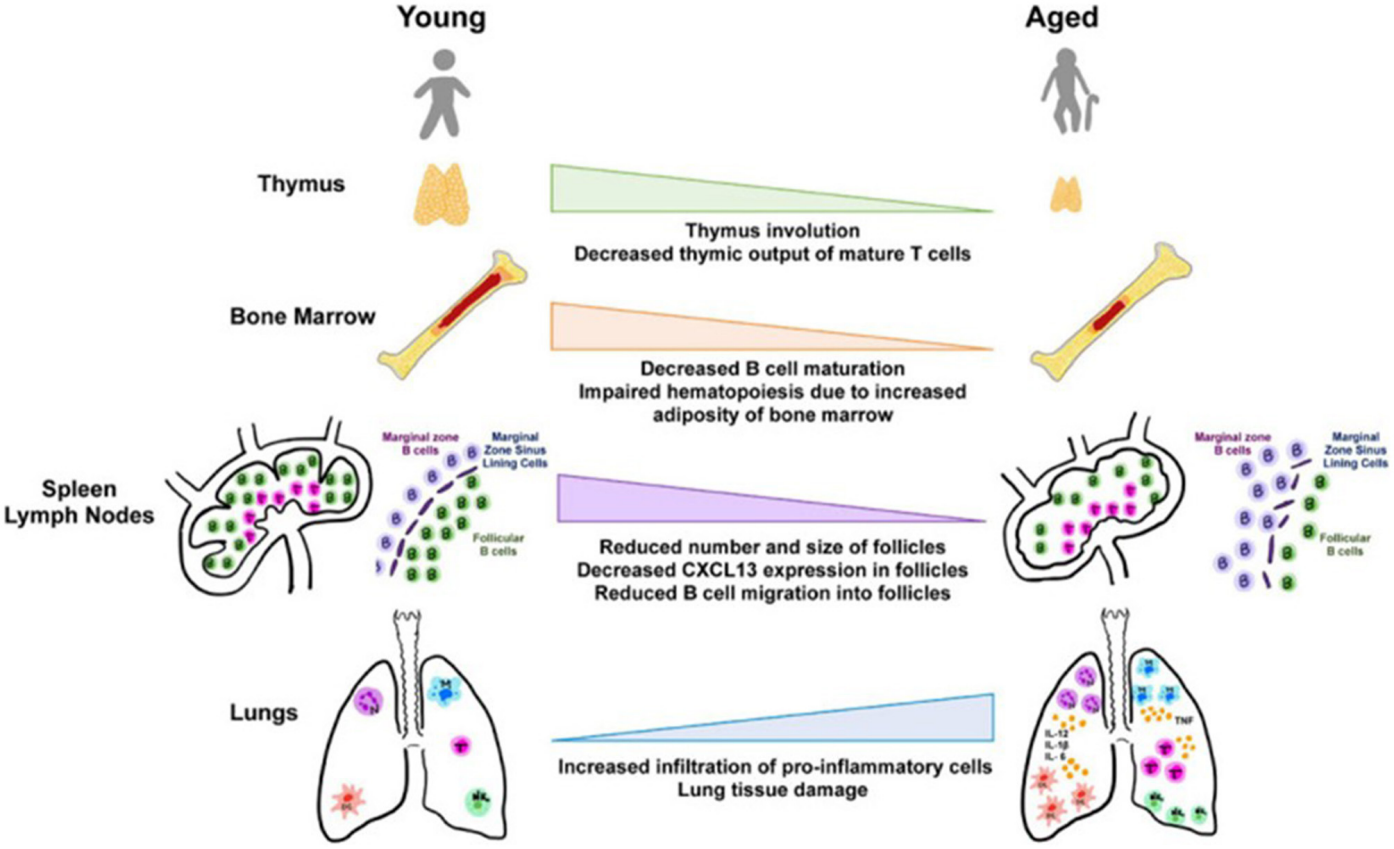
Aging and tissues in immunity (23).

Furthermore, Figure 2 revealed that primary lymphoid organ degeneration reduces the production of naive B and T cells, resulting in decreased migration to antigen encounter regions and secondary lymphoid organs. In addition, proinflammatory mediators can accumulate in the lungs and extrapulmonary organs (22).

## Aging and Innate Immunity

The host's initial line of defense against infectious diseases is the innate immune system (24). Neutrophils, natural killer (NK) and natural killer T (NKT) cells, monocytes/macrophages, and DCs are all part of the innate immune system, which mediates the first interactions with pathogens. These types of cells have age-related defects in their activation, which are associated with compromised signal transduction cascades, such as the Toll-like Receptors (TLRs) (25). Multiple studies suggested that aging has a deleterious impact on the functions of innate immunity (26). Moreover, in non-healthy aged individuals, innate immunity is considerably weakened, and this impairment makes a significant contribution to a decline in overall immune responses as well as increased morbidity and mortality in the elderly due to infections (27). According to several studies in human and animal models, TLR expression and function decrease with age. In aged DCs, mitochondrial membrane potential, ATP turnover and coupling efficiency, and baseline oxidative phosphorylation were found to be decreased. Moreover, an increase in proton leak and reactive oxygen species (ROS) production were observed in aged DCs (16). Hence, DC appears to be functionally impaired in aging when it comes to antigen uptake, and phagocytosis of apoptotic cells. Other DC elements that change with age are associated with TLR function (inappropriate persistence of TLR activation in specific systems), antigen processing, and cell migration, which is linked to a change in the phosphoinositide 3 kinase pathway (28). Interleukin (IL)-15 interferon-alpha (INF-α) and tumor necrosis factor alpha (TNF-α) levels have also been found to be lowered in aged people. Furthermore, a study demonstrated that older mice's DCs have an inadequate ability to stimulate CD8^+^ T cells (29). Reduced TNF- production and lower DC maturation have been linked to impaired influenza-specific CD8^+^ T cell response in elderly people. While neutrophils and macrophages are phagocytic cells of first-line defense, their phagocytic functions diminish with advanced aging which contributes to chronic low-grade inflammation and dysregulation of macrophage-mediated immunosuppression. Because of the constant activation of the PI3K cascade in older people, neutrophils migrate incorrectly and spread further in response to stimuli, have lower phagocytosis, and have lower intracellular killing activity. Impaired anti-apoptotic responses to GM-CSF facilitated by JAK-STAT tyrosine kinase and PI3K-AKT cascades are the primary causes of age-related defects in neutrophils (30). The aged non-hematopoietic environment plays a key role in NK cell maturation and function. Increased reactivation rates of latent Mycobacterium tuberculosis, decreased inflammatory responses, and an increase in bacterial and fungal infections are all linked to changes in NK cell biology that occur with human aging (16).

In view of these facts, studies of the innate immune system in aged individuals who are not influenced by systemic diseases that could affect the immune system, as well as studies that use medications that could interfere with immunological parameters, could provide information primarily on the intrinsic impact of aging on immune responses (31). In contrast, studies on unhealthy elderly people with extrinsic factors like chronic infections, cancer, malnutrition, inflammatory diseases, and neurological conditions are biased. However, studies on innate immunity should be encouraged not only in healthy elderly but also in unhealthy and frail elderly people, as these people are at a higher risk of infection and related complications (32). Since innate immunity is the first line of defense against pathogens, increasing innate immunity in the elderly could be a viable option for fighting infection and improving the quality of life, providing that inflammatory diseases are not exacerbated (33).

## Lung Complications and Their Mortality Rates With Respect to Age

### Pneumonia

Pneumonia is an infection that causes inflammation of one or both lungs. The lungs are comprised of small sacs known as alveoli which are the primary site of gaseous exchange. When a healthy person breathes, their alveoli are filled with air, but in individuals suffering from pneumonia, the alveoli are filled with fluid and pus which results in breathing difficulty due to a limited amount of oxygen (22, 34). TLR4 expression, which is important for the response to *Streptococcus pneumoniae*, is lower in the elderly and during infection. In mice, aging has been shown to decrease CD8 T-cell diversity as well as the immune response to influenza virus infection. The number of naive and memory T-cells decreases as people age (35).

**Figure 3:**
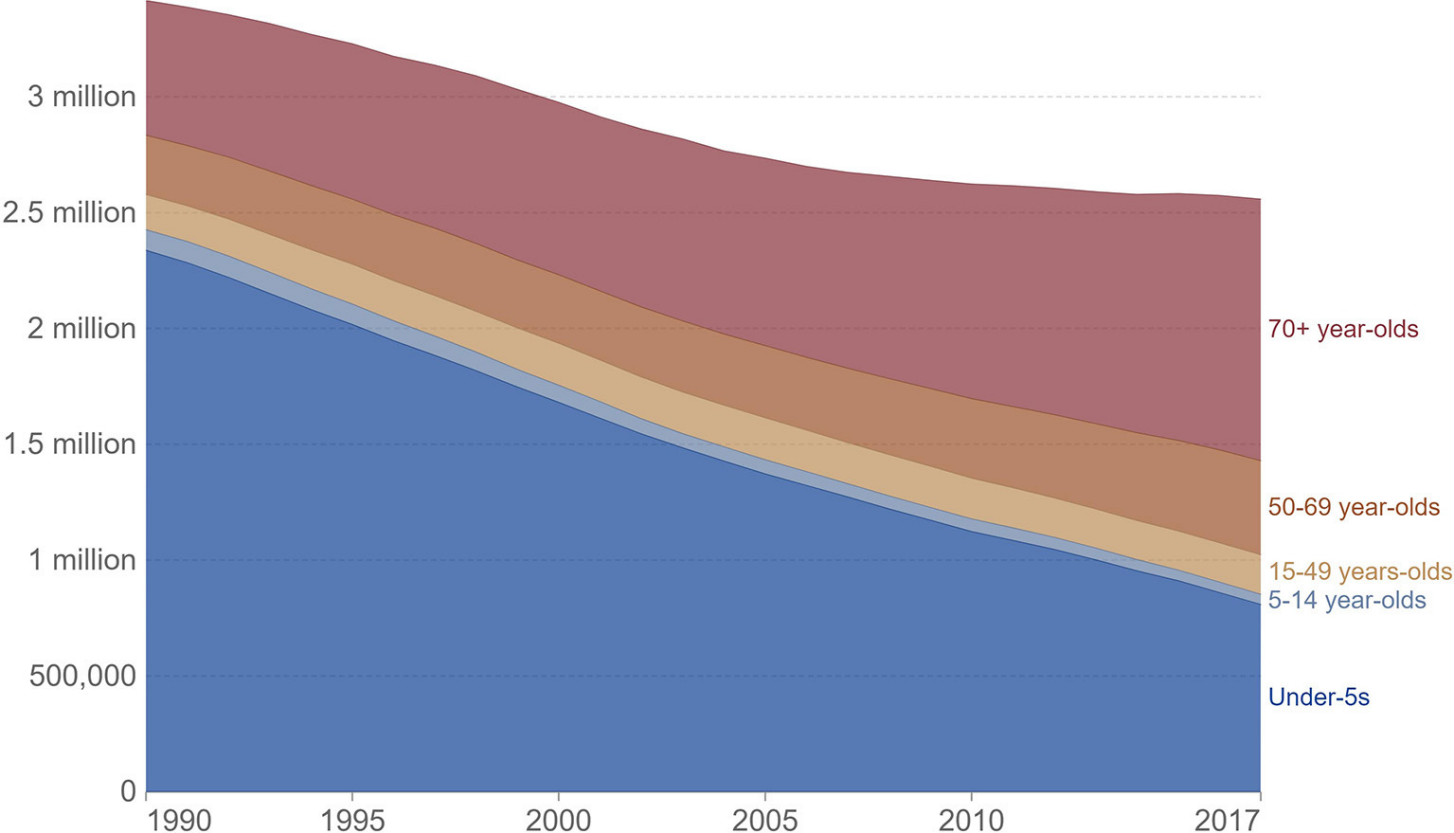
IHME, global burden of disease study (GBD), OurWorldInData.org/pneumonia.

More than 2,000 patients (aged ≥ 65 years) were analyzed by Conte et al., which revealed that age ≥ 85 years was considerably linked with an increased mortality rate (2, 36). In Europe, death rates for pneumonia were considerably elevated in children (up to the age of four) and adults (aged 75 and over) relative to other age groups. Pneumonia mortality rates were greater among the elderly in Western Europe (279 deaths per 100,000 people) (3). A study reported in 2017 revealed an elevated mortality rate among aged people (70 and older) suffering from pneumonia and in this age group, around 261 patients (out of 100,000 people) were died due to pneumonia, as indicated in Figure 3.

### Idiopathic Pulmonary Fibrosis

Idiopathic pulmonary fibrosis (IPF) is a deadly lung disease with unknown reasons. IPF is an irreversible disease that is considerably associated with aging. The mechanism regarding IPF pathogenesis is not clearly understood and up to date, there is no specific treatment for this disease (37).

Among the elderly population, the quality of life was decreased in patients suffering from IPF. Moreover, the reported studies have also been indicated that in aged people, the majority of IPF patients suffer from comorbidities (38). IPF is characterized by abnormal activation of alveolar epithelial cells, fibroblasts and myofibroblasts accumulation, and excessive extracellular matrix (ECM) formation (39). Furthermore, telomeres in lung epithelia and peripheral blood cells are shortened in patients with IPF. Programmed cell arrest (senescence) and apoptosis occur when telomeres reach a critical length. Patients with IPF have abnormal cellular senescence, particularly in bone marrow-derived stem cells including fibrocytes (40).

**Figure 4:**
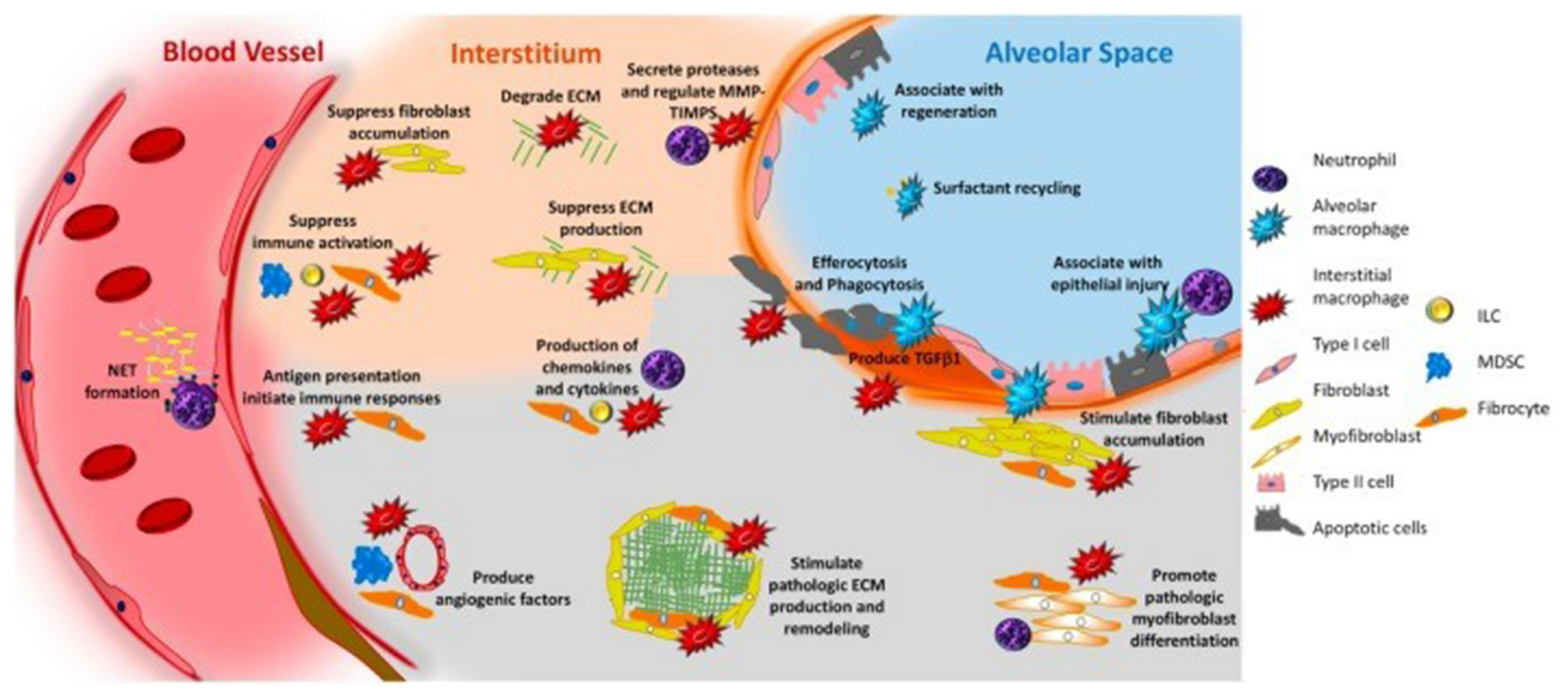
Immune system and pulmonary fibrosis (41).

Reported studies have been revealed that aging immunity considerably contributes in the progression of fibrosis. Macrophages, both alveolar and interstitial can develop fibrosis-modifying features in response to exposure with pathogen-associated molecular patterns or danger-associated molecular patterns, or activation with different mediators and results in the production of TGFβ1and soluble mediators that cause accumulation and activation of fibroblasts, generation of TIMPS, and MMPS which involved in the remodeling of ECM, formation of angiogenic factors, secretion of lipid mediators, and regulation of stem cell renewal and structural cell injury. Neutrophils produce NE, TIMPS, and MMPs, which determine whether ECM aggregates or degrades. Neutrophils also play a role in the creation of neutrophil extracellular traps, which may induce fibrosis by releasing TGF1 and activating myofibroblasts. Circulating fibrocytes are mesenchymal cells generated from bone marrow that enter the lungs through the bloodstream. They perform a variety of functions in the lung, including differentiation into myofibroblasts and fibroblasts, ECM formation, wound contraction, antigen presentation, production of cytokines and chemokines, and production of soluble mediators regulate the angiogenesis-related pathways. Immunosuppressive cells known as myeloid-derived suppressor cells (MDSC, blue) have been linked to ECM remodeling and pulmonary hypertension. Innate lymphoid cells secrete cytokines that may play a role in the regulation of fibroblast accumulation and ECM formation. In Figure 4, the roles of each cell are displayed in a typeface that matches the cell's color.

Furthermore, IPF is linked to a poor prognosis, while the mortality rates of patients associated with IPF rise with aging and are substantially greater in males than in women (42). According to the reported survey, the estimated mean survival rate for IPF was determined to be 2–5 years from the time of diagnosis (43). Men died at a rate of 64.3 deaths per million, while women died at a rate of 58.4 deaths per million (44).

### Coronavirus Disease-2019 (COVID-19)

The COVID-19 pandemic, caused by the novel coronavirus severe acute respiratory syndrome coronavirus 2 (SARSCoV2), had wreaked havoc on the world as of early December 2020, with more than 72 million morbidities and 1.6 million deaths. The aged group has a disproportionately high number of illnesses and deaths. Several cases of pneumonia with unclear etiology were reported in Hubei, China in December 2019, and were later identified as COVID-19 caused by SARS-CoV-2. The virus uses angiotensin-converting enzyme 2 (ACE2) receptors to enter the body. ACE2 is a part of the renin-angiotensin-aldosterone system and is expressed in the lower respiratory tract along the alveolar epithelium. The reported studies have revealed that aging has been linked to a decrease in ACE2 expression. A reduction in ACE2 receptor expression, in combination with aging-related immune inflammation and comorbidities, may compromise the anti-inflammatory response and predispose older people to enhance inflammatory responses, which is one of COVID-19's hallmarks (45). Aging impairs immune cell migration and signaling downstream of pattern recognition receptors (PRR) activation, resulting in increased cytokine secretion and dysregulation. In innate immune cells, age-related changes in TLR protein expression can trigger the production of cytokines. An elevated level of cytokines reflects high basal TLR activation, which cannot be further activated in response to a pathogen, resulting in innate immune response failure (46). Because IFN production is impaired in COVID-19 patients, there is an imbalance between pro-inflammatory and pro-repair airway macrophages. Antigen-presenting cells become functionally impaired as a result of age-related reductions in IL-12 production by DCs and changes in the microenvironment caused by changes in the splenic marginal zones (47). Changes in the pulmonary microenvironment affect DC maturation and migration to lymphoid organs, affecting T cell activation in COVID-19 patients (45).

**Figure 5:**
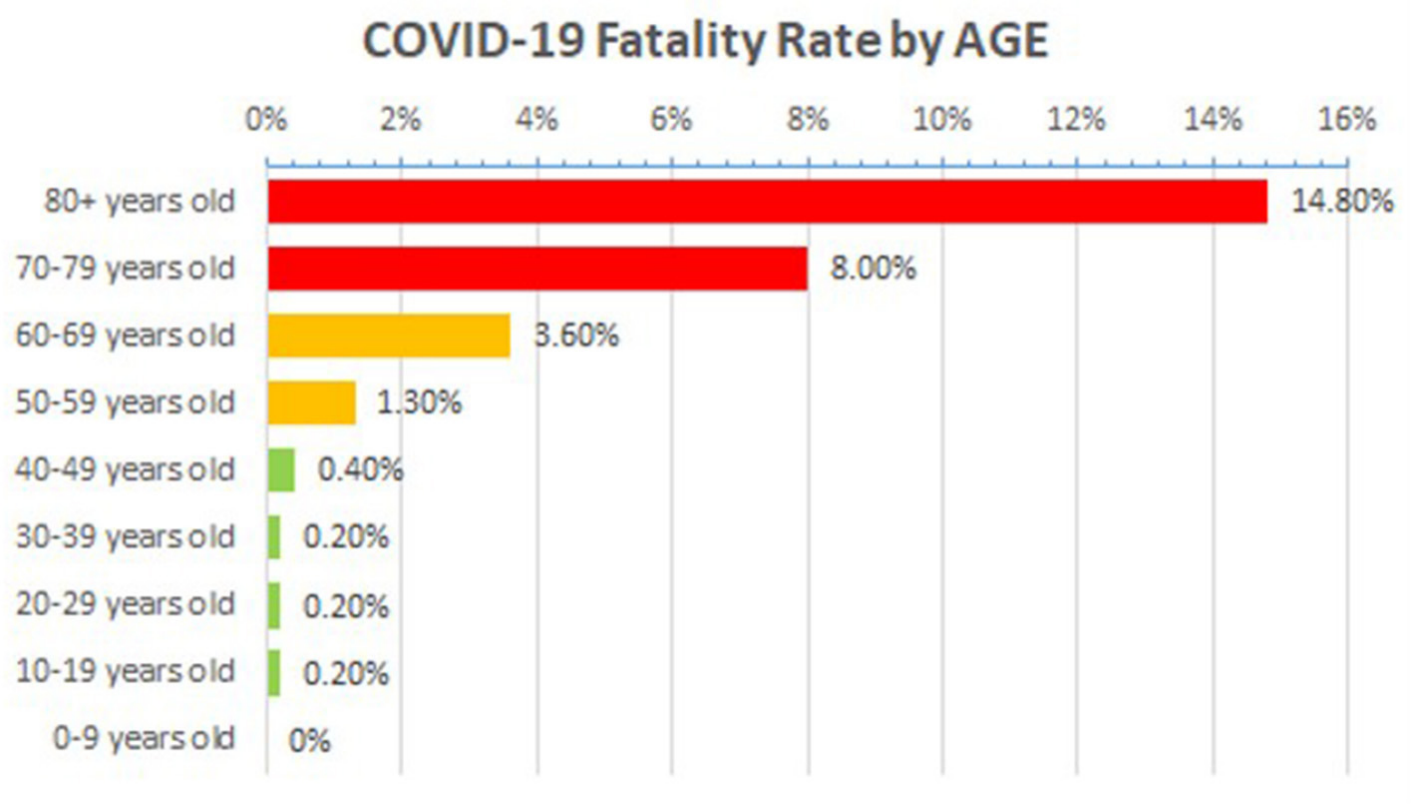
Age-associated mortality rate in COVID-19 patients across the globe, Source data: https://www.worldometers.info/coronavirus/coronavirus-age-sex-demographics/ (accessed March 13, 2020).

There were no noticeable variations in viral load between symptomatic and asymptomatic individuals (7), resulting in global dissemination and an underestimated death ratio (4). Furthermore, the virus affects people of all ages, but those with co-morbidities or who are older have a significantly higher rate of morbidity and mortality (5, 6). Patients with co-morbidities of Ischemic Heart Disease, hypertension, and diabetes had increased odds of fatal cases in China, according to an analysis of fatal cases. However, no evidence of a greater risk among pregnant women has been found (12). According to the literature, the death rate among hospitalized patients was 15%, with an average time from onset of symptoms to death of 14 days (13). Based on the data collected from Italy and China, Corona patients have a mortality rate of 2.3%, with more than half of the mortality reported in patients with age 50 years or older (14). Case mortality was 36% in patients at an age of 64 years or older in the largest published series from Northern Italy (15). The global mortality rate from COVID-19 approaches 15% at age 80 (48), as represented in Figure 5. In the given figure, the rat**e** of mortality was calculated by the following formula;


$$\begin{array}{l} \text{Mortality rate}=\left(\text{number of deaths}/\text{number of cases}\right)=\\ \text{probability of dying if infected by the virus }\left(\text{\%}\right). \end{array}$$

### Asthma

**Figure 6:**
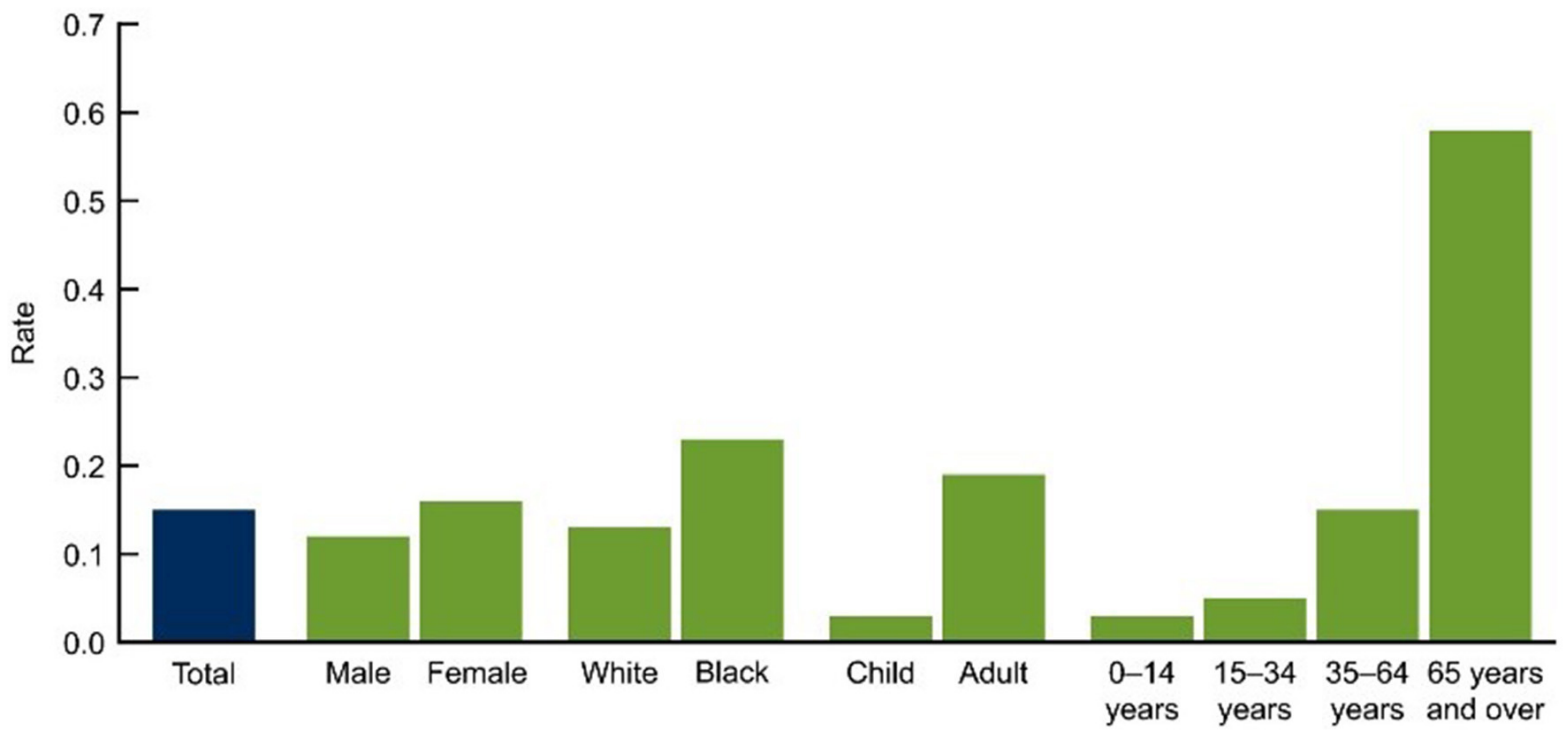
Asthma deaths per 1,000 by demographic (2007–2009) (49). CDC/NCHS, National Vital Statistics System, and National Health Interview Survey Data for table available online at: http://www.cdc.gov/nchs/products/databriefs/db94.htm.

Asthma is the most common disease which affects around 300 million people (all age groups) across the globe. However, the diagnosis, as well as management of asthma is very difficult in aged people because of physiological and immunological variations associated with age (17). In spite of recent considerable enhancements in the treatment of asthma, there is still a need for further improvement in care and patient outcomes, especially in elderly patients suffering from asthma. Elderly individuals in their middle years have the highest incidence, yet, the mortality ratio is higher in the elderly. The underlying pathophysiology of asthma in older individuals, as well as the best ways to treat it, have long been disregarded. Aged asthma patients have the highest rates of morbidity and mortality, as described in Figure 6. The etiology of asthma in older adults is complicated since it is influenced by decline immune functions associated with aging as well as changes in inflammation (18).

**Figure 7:**
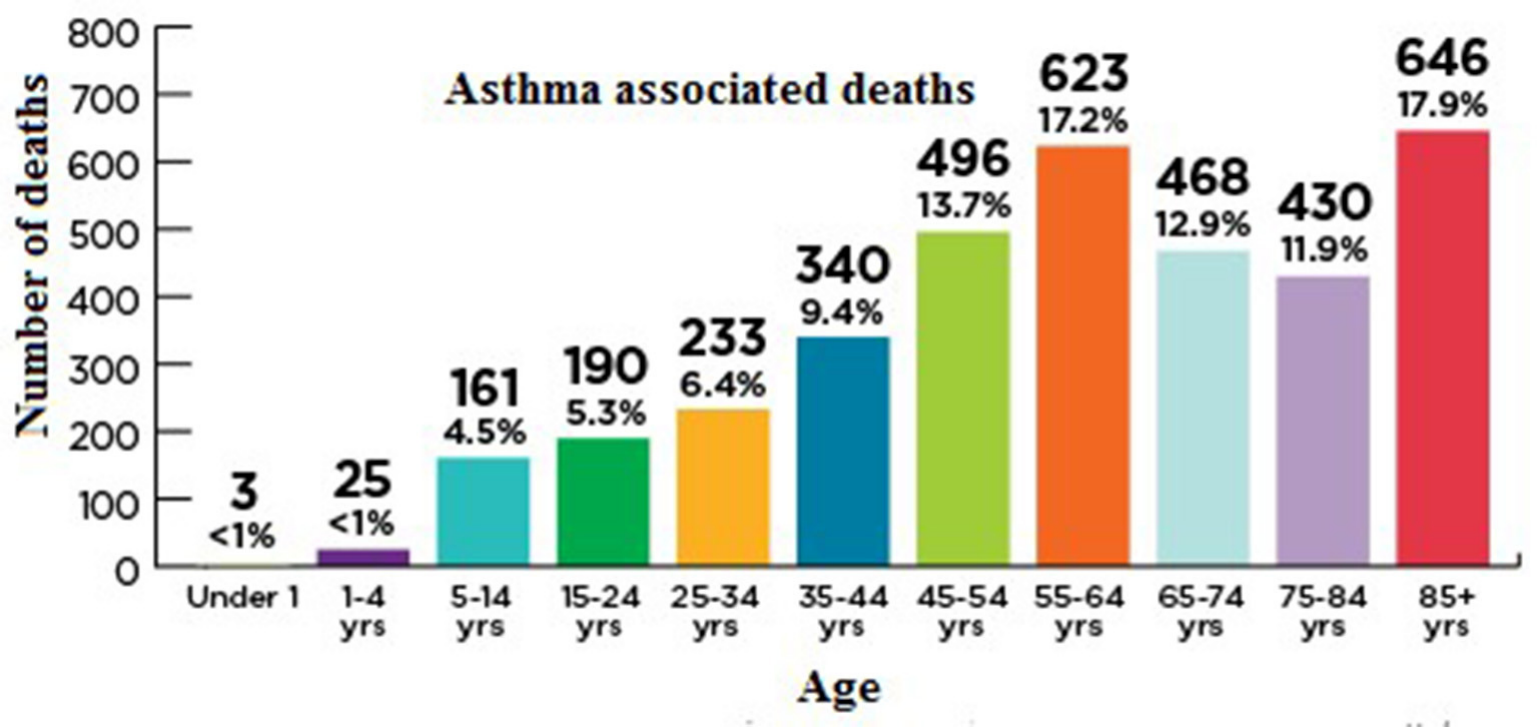
Age-associated mortality rate in asthma patients. Asthma and Allergy Foundation of America asthmacapitals.com.

In 2015, 3,615 people (10 people/day) were died because of asthma in the united states. The death rate has been elevated in the elderly, as depicted in Figure 7 and as people get older, their chances of dying from asthma rises because they are probably misdiagnosed, undertreated, and are managing several health complications with a weak immunity (52).

The mechanism underlying asthma is unknown. However, it has been suggested that it may occur as a result of viral infection that promotes persistent inflammatory change when coupled with the effects of immunosenescence. Antibody production may be decreased in the aged, resulting in elevated antigen persistence and specificity. T-cell population shifts, varied B-cell antigen processing, and eosinophil function coupled with a decrease in phagocytic potentials are all caused by thymic involution and all these events contribute to a distinct immunological environment in older asthmatic patients (53). Furthermore, T-cells activation has been elevated in the elderly, with overexpression of human leukocyte antigen (HLA-) DR and CD69. An elevation in airway neutrophils has also been seen in older asthma patients, implying that asthmatic phenotypes varied as people age. These findings suggest a possible link between immunosenescence and asthma. However, this relationship has not yet been fully established (54).

### Chronic Obstructive Pulmonary Disease (COPD)

COPD is a chronic respiratory condition marked by persistent respiratory symptoms and restricted airflow. COPD has a significant influence on public health, owing to its rising prevalence, morbidity, and mortality rates (55). COPD's prevalence and burden are likely to rise in the next decades as a result of ongoing exposure to risk factors and the global population's aging, and it is expected to become the world's third leading cause of death by 2020 (56, 57). COPD morbidity and mortality rates are higher as people get older. The morphological and functional resistance of the respiratory system deteriorates with age, resulting to a rise in morbidity. Individuals aged 60–79 years have a higher rate of respiratory morbidity and a lower FEV1/FVC ratio, according to the European Lung White Book (58).

**Figure 8:**
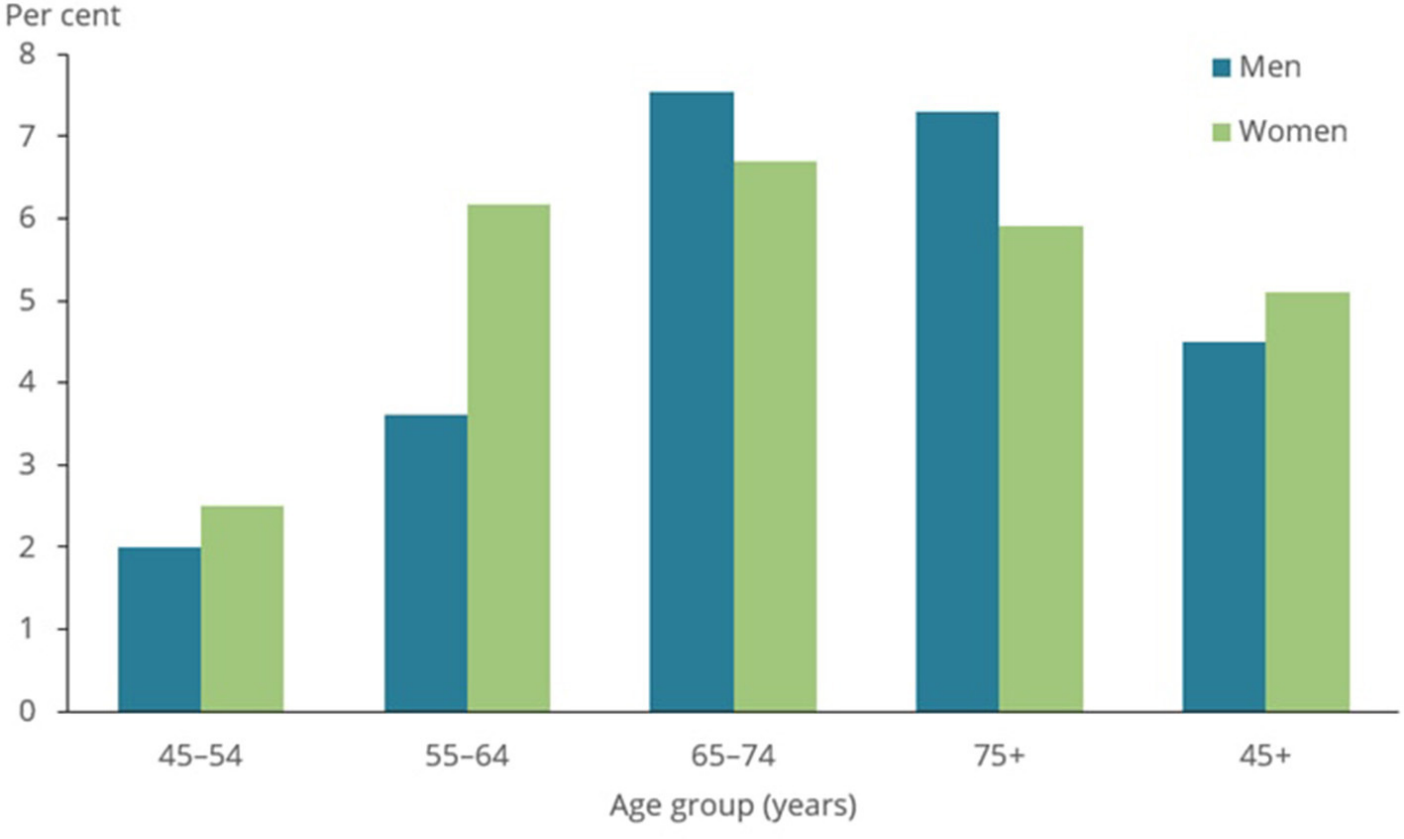
Prevalence of COPD among people aged 45 and over, by sex and age group, 2017–18 (50).

The lung function of approximately 10,000 participants was examined in the Burden of Obstructive Lung Disease (BOLD) study (59). Spirometry testing, as well as questionnaires regarding respiratory symptoms, health status, and exposure to COPD risk factors, were used in the BOLD project to assess the prevalence of COPD. BOLD estimated that 10% of adults aged 40 and over in 12 nations (including Australia, China, Turkey, Iceland, Germany, the United States, and Canada) have COPD. As shown in Figure 8, the COPD prevalence was found to be 7.5% for those aged 40 and over and 30 percent for people aged 75 and over in later research conducted in Australia using a technique that closely resembled that employed in the worldwide BOLD study (60).

There is evidence that aging and COPD have multiple pathways and processes in common. Individuals with COPD have a suppressed innate immune system, making them more susceptible to infections and malignancies (61). Aging is linked to diminished epithelial barrier function (62), cilia structure and function defects (63), and reduced generation of antimicrobial and anti-inflammatory peptides by epithelial cells, such as SLPI (64). Elevated numbers of phagocytes, such as macrophages, monocytes, and neutrophils, are linked to both COPD and aging. There is also a decrease in host defensive mechanisms such as macrophage phagocytosis, inefficient chemotaxis, diminished neutrophil bactericidal function, and altered dendritic and natural killer cell capabilities. Inflamm-aging is commonly linked to immunosuppression and low-grade inflammation (65). Subsequent inflammation develops when secondary lung infection occurs as a result of a weakened host response (66). Immunosenescence promotes telomere shortening in COPD patients' leukocytes regardless of smoking status, as well as an increase in inflammatory markers, suggesting that disease-specific variables may contribute to immune cells' premature aging (67).

### Lung Cancer

Lung cancer is a deadly respiratory disease that mostly affects older people. Around 50% of lung cancer has been diagnosed in patients aged ≥70 years while around 14% has been observed in patients older than 80. Physiological as well as medical features of elderly patients (suffering from cancer) make the choice of their better treatment more challenging. As discussed in the above respiratory complications, the increasing incidence of lung cancer is also significantly linked with the age-associated decline in immune functions. NSCLC (Non–small cell lung cancer) is the most common form of lung cancer, accounting for around 85% of cases (68). Lung cancer incidence and prevalence have increased in the elderly population, demonstrating a link between aging and the development of cancer. Oncogenes become more active and tumor suppressor genes become inactive when cells are damaged by either free radicals or viruses (69). Age-related immune adaptations play a role in the development of cancer. The immune stimulation of T-cells by DCs, for example, is critical for their activation under normal circumstances, but this is altered as people age. The link between T-cell immunosenescence and terminal differentiation, as well as the decrease in circulating naive CD8^+^ lymphocytes in senescent patients, suggests that the immune system's ability to respond to tumor antigenic diversity and emerging neoantigens may be impaired in T-cell immunosenescence (69). TLR signaling becomes less effective as people get older, resulting in a variety of abnormal responses and phagocytic dysfunction. Ineffective neutrophil and macrophage function in the elderly also contributes to the development and progression of tumors (70).

**Figure 9:**
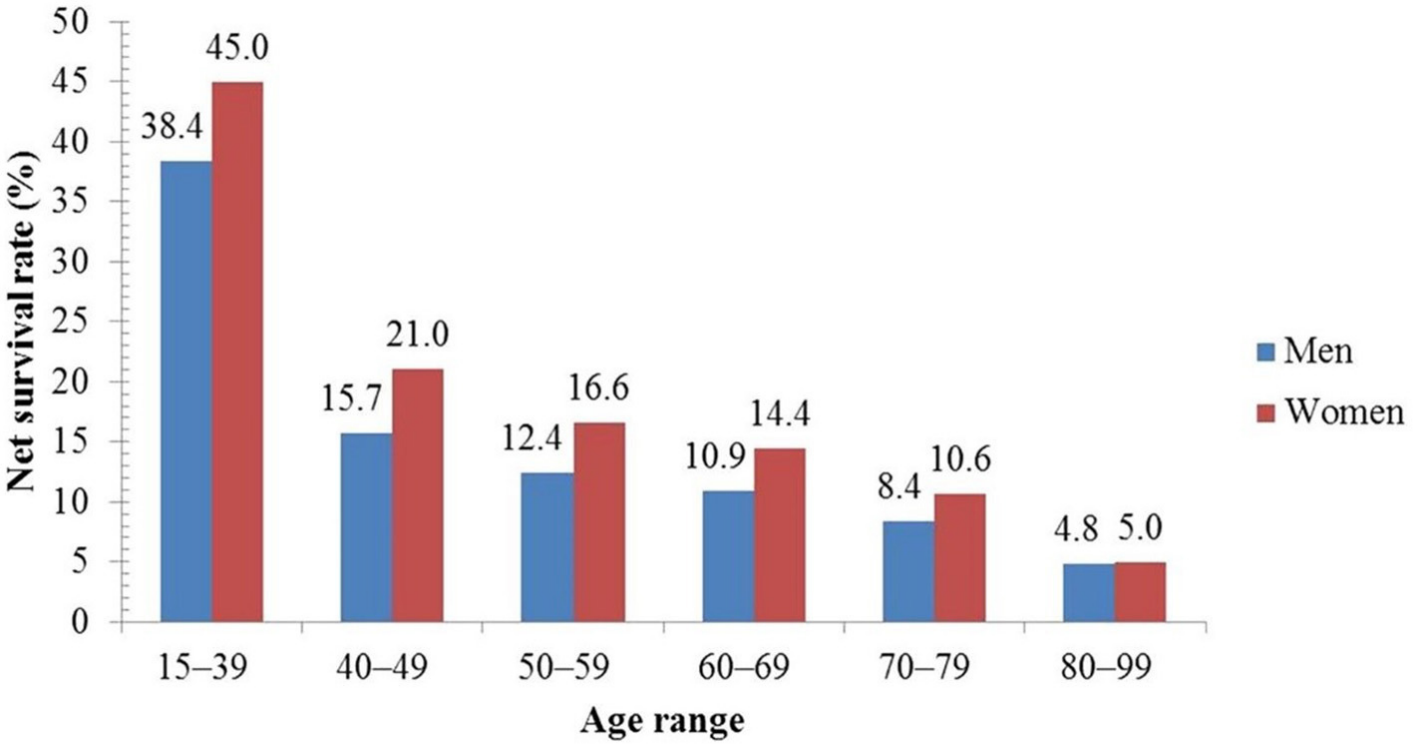
Five-year net survival rate of lung cancer patients by age in the UK (51).

Reported studies revealed that <0.5% of deaths (associated with lung cancer) occur at an age <40 years (71) while an elevated rate of incidence has been reported elderly population. In the UK (United Kingdom), each year around 6 in 10 cases (61%) of lung cancer are diagnosed in people aged ≥70 years (72). Up to the age of 39, both men and women have a 0.03% chance of having lung cancer (71). The incidence rates begin to rise sharply at the age of 45–49, peaking in the male and female age groups of 85–89 and 80–84, accordingly. In the US, the median age of diagnosis is 70 years, and 68% of patients are diagnosed after the age of 65 (71) and 14% of lung cancers are detected in individuals over the age of 80 (71). Another study from the UK found that between 2007 and 2011, the 5-year age-standardized net survival rates for males with lung cancer lowered from 38.4 to 4.8%, and for women from 45.0 to 5.0%, as shown in Figure 9. This study revealed that 5-year survival (in lung cancer) is elevated in the youngest men and women and diminishes with increasing age (51, 73).

## Conclusions

The findings we summed up in the current review revealed that aging hallmarks are the key variables associated with the high mortality rates of severe respiratory diseases, such as pneumonia, coronavirus infection, asthma, COPD, lung cancer, IPF, and acute lung injury. These accumulating evidences also suggest that age is a cause of many diseases. Our study will help the future research of physiological variations that occur with age and, more importantly, their impact on respiratory illnesses that are the major cause of morbidity and mortality in the elderly.

## Author Contributions

MP: review design. YL: data collection. CW: draft manuscript preparation. All authors reviewed and approved the final version of the manuscript.

## Conflict of Interest

The authors declare that the research was conducted in the absence of any commercial or financial relationships that could be construed as a potential conflict of interest.

## Publisher's Note

All claims expressed in this article are solely those of the authors and do not necessarily represent those of their affiliated organizations, or those of the publisher, the editors and the reviewers. Any product that may be evaluated in this article, or claim that may be made by its manufacturer, is not guaranteed or endorsed by the publisher.
